# Stance Phase Detection by Inertial Measurement Unit Placed on the Metacarpus of Horses Trotting on Hard and Soft Straight Lines and Circles

**DOI:** 10.3390/s22030703

**Published:** 2022-01-18

**Authors:** Chloé Hatrisse, Claire Macaire, Marie Sapone, Camille Hebert, Sandrine Hanne-Poujade, Emeline De Azevedo, Frederic Marin, Pauline Martin, Henry Chateau

**Affiliations:** 1 Ecole Nationale Vétérinaire d’Alfort, USC INRAE-ENVA 957 BPLC, CWD-VetLab, 94700 Maisons-Alfort, France; claire.macaire@vet-alfort.fr (C.M.); emeline.de-azevedo@vet-alfort.fr (E.D.A.); henry.chateau@vet-alfort.fr (H.C.); 2 LIM France, Chemin Fontaine de Fanny, 24300 Nontron, France; msapone@lim-group.com (M.S.); chebert@lim-group.com (C.H.); shannepoujade@lim-group.com (S.H.-P.); pmartin@lim-group.com (P.M.); 3 Université de Technologie de Compiègne (UTC), UMR CNRS 7338 BioMécanique et BioIngénierie, Alliance Sorbonne Université, 60200 Compiegne, France; frederic.marin@utc.fr

**Keywords:** horse, locomotion, stance phase, gait events, inertial measurement units

## Abstract

The development of on-board technologies has enabled the development of quantification systems to monitor equine locomotion parameters. Their relevance among others relies on their ability to determine specific locomotor events such as foot-on and heel-off events. The objective of this study was to compare the accuracy of different methods for an automatic gait events detection from inertial measurement units (IMUs). IMUs were positioned on the cannon bone, hooves, and withers of seven horses trotting on hard and soft straight lines and circles. Longitudinal acceleration and angular velocity around the latero-medial axis of the cannon bone, and withers dorso-ventral displacement data were identified to tag the foot-on and a heel-off events. The results were compared with a reference method based on hoof-mounted-IMU data. The developed method showed bias less than 1.79%, 1.46%, 3.45% and −1.94% of stride duration, respectively, for forelimb foot-on and heel-off, and for hindlimb foot-on and heel-off detection, compared to our reference method. The results of this study showed that the developed gait-events detection method had a similar accuracy to other methods developed for straight line analysis and extended this validation to other types of exercise (circles) and ground surface (soft surface).

## 1. Introduction

Musculoskeletal disorders are the first reason for medical consultation in horses [1]. They lead to low performance and all are signs of pain and loss of well-being [2]. Competitions involve high-intensity training for horses and cause micro-to-large lesions on the horse bone or muscles [3,4]. Consequently, monitoring horse locomotion can be considered as a priority and as a major concern for the improvement of animal welfare.

Gait symmetry degradation is a marker of an injured horse [5,6], which is brought to the fore by spatiotemporal differences between both bipeds in a movement. On a treadmill, an increase in the stance duration could be an indicator of forelimb lameness [7]. Foot-on and heel-off are spatiotemporal events essential to calculate locomotor parameters, and especially the stance duration [8,9]. Commonly, the stance phase of one leg is defined as the moment between the hoof being completely laid flat on the ground (foot-on) to the take-off of the heel (heel-off) [10,11]. These events can be examined visually, but the temporal resolution of the human eye limits its precise distinction [12]. Several methods based on force plate [13,14] and optical motion capture (OMC) [15,16] analysis are available to monitor these spatiotemporal events. Although these methods are highly reliable, they require laboratory conditions with limited exercise conditions. These conditions do not reflect the full spectrum of exercises needed by the clinician during a complete locomotor examination [17].

Recently, it was demonstrated that inertial measurement units (IMUs) are a real alternative to performing motion capture [18]. The use of IMUs allows recording the horse locomotion with on-board technologies and in field conditions [19,20]. It was also demonstrated that IMUs achieved precision and accuracy as well as a force plate [19] or OMC system [20,21,22] to quantify locomotion.

However, the use of IMUs for motion capture required a specific detection algorithm [8,9]. Studies investigated the horse gait events detection accuracy of IMUs mounted on the lateral quarter of the horse hoof. Thanks to this IMU location, they determined all the stance phase events, from the first hoof impact to complete take-off [10,11]. This protocol using IMUs mounted on the horse hoof is considered a reference method to detect foot-on and heel-off events [10,11]. However, in routine investigation, the fixation on the hoof is critical because IMUs have to be glued on or screwed to the hoof. An alternative localization of the IMU could be on the cannon bone, with the advantage of being easily integrated into boots [8].

Previous studies investigated gait event detection methods from an IMU positioned on the cannon bone at trot, on a treadmill, compared to a gold-standard method based on an OMC system [8], and on different grounds, only in straight lines [9]. However, no methods dedicated to circle exercises or different ground surfaces have yet been addressed.

In this context, the aim of this study is to test a method for automatic foot-on and heel-off event detection by an IMU mounted on the horse cannon bone, in straight lines and circles on different surfaces (hard and soft materials).

## 2. Materials and Methods

### 2.1. Horses

Seven sound horses of trotting and thoroughbred breeds (four geldings and three mares, height 162 ± 3 cm (mean ± SD), age 13 ± 4 years (mean ± SD)) were included in the study. Prior to the procedure, the protocol was examined and approved by the dedicated ethics committee on animal investigation (Comité National de Reflexion Ethique sur l’Experimentation Animale, Anses/ENVA/UPEC n°HE_2017_01).

### 2.2. Data Acquisition

**Figure 1:**
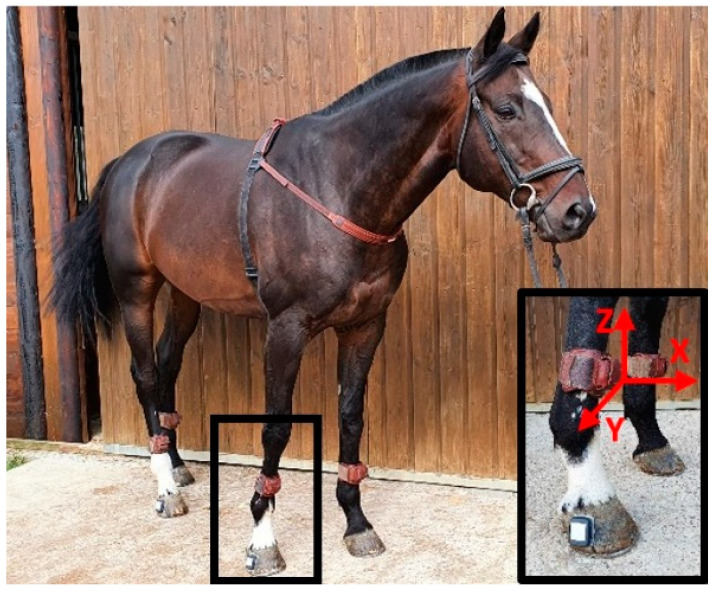
IMUs localization on horse: on the withers, on the four limbs, in the center of the dorsal side of the third metacarpal bone (cannon bone), and on the lateral quarter of the right front and hind hoof.

Horses were equipped with seven synchronized IMUs (Figure 1) (ProMove-mini, Inertia Technology BV, Enschede, the Netherlands) with a full-scale range of 16 g, 2000°/s, 16 bits, sampling at 200 Hz. IMUs were positioned on the withers and in the center of the dorsal side of the third metacarpal bone (cannon bone) of the four limbs, and the last two IMUs were mounted on the lateral quarter of the right front and hind hoof of the horse (Figure 1).

The withers IMU was used to help us detect the trot gait. The four cannon bone IMUs were used to develop and test our foot-on and heel-off events detection methods. The hoof IMUs were used to obtain the foot-on and heel-off event references. Unfortunately, we did not have more IMUs available to equip the four horse hooves. The presented results were therefore only focused on the right limbs.

Two different exercises were realized, at trot, in straight lines and circles. Two types of ground were tested: hard (asphalt or rubber) and soft ground (sand). Sessions included: four straight lines of 25 m on asphalt ground, three left-circles and three right-circles of 14 m diameter on rubber ground (considered as hard ground), six straight lines of 30 m on sand, and finally, three left-circles and three right-circles of 14 m diameter on sand. An average of 42 ± 4 strides (mean ± SD) per horse were collected for each condition. On the left circles, the IMU equipped limb (right limb) was outside the circle. On the right circles, the IMU equipped limb was inside the circle.

### 2.3. Data Processing

**Figure 2:**
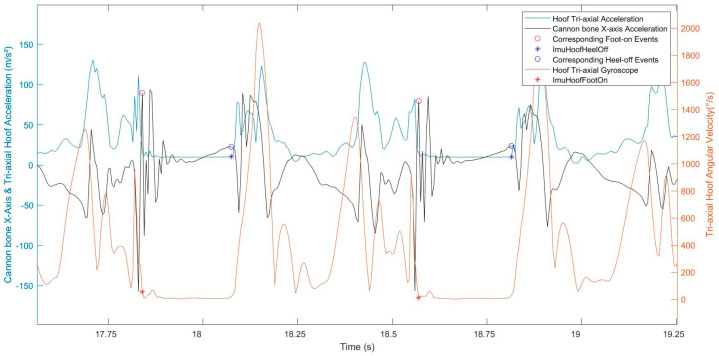
Representation of the normalized signals (normalized by the maximum value) of the hoof tri-axial acceleration (blue curve), the hoof tri-axial gyroscope (red curve), and the cannon bone *x*-axis acceleration (black curve) of a horse trotting on straight line and hard ground. The red stars correspond to the reference foot-on and the blue stars correspond to the reference heel-off. The cannon bone *x*-axis acceleration at these same events also appear on the cannon bone curve (blue and red circles on the yellow curve).

Following locomotion data acquisition, the first step was to detect the foot-on and heel-off events from both of the hoof-mounted IMUs, based on the methods developed by Tijssen & al. (2020) [10,11]. The reference foot-on, obtained from the hoof IMU data, matched with the beginning of the plateau on the hoof tri-axial gyroscope curve [10]. The reference heel-off matched with the end of the plateau [10,11] on the hoof tri-axial acceleration curve (Figure 2). This first step was used as a reference for our study.

**Figure 3:**
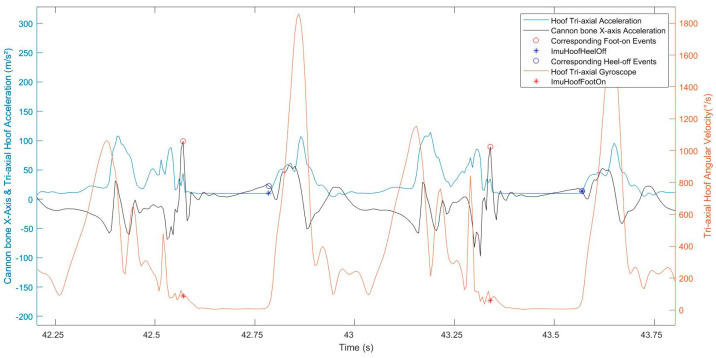
Representation of the normalized signals of the hoof tri-axial acceleration (blue curve), the hoof tri-axial gyroscope (red curve), and the cannon bone *x*-axis acceleration (black curve) of a horse trotting in a straight line on soft ground. The red stars correspond to the reference foot-on; the blue stars correspond to the reference heel-off. The cannon bone *x*-axis acceleration at these same events also appears on the cannon bone curve (blue and red circles on the yellow curve).

The methods developed by Tijssen & al. (2020) [10,11] were tested and validated by themselves on hard ground. We supposed that they were extendable to soft surfaces, so the same reasoning was applied. The beginning of the plateau, just after the last noticeable peak, on the hoof tri-axial gyroscope curve was also chosen for the foot-on event on soft ground (Figure 3). After the foot-on event, a decrease in the hoof tri-axial gyroscope curve could be noticed, which could match with the hoof sinking into the sand. The hoof tri-axial acceleration curve shape did not change between hard and soft ground surfaces; the choice of heel-off on soft ground was not different.

According to the exercise (straight line: “Line” or circle: “CircleIn” when the IMU equipped limb was inside the circle, and “CircleOut” when the IMU equipped limb was outside the circle), the ground condition (asphalt/rubber: “Hard” or sand: “Soft”) and the limb (forelimb: “Fore” or hindlimb: “Hind”) timing of the *i*th stride of the foot-on and heel-off was labeled ImuHoofFootOn (*i*, “exercise”, “ground”, “limb”) and ImuHoofHeelOff (*i*, “exercise”, “ground”, “limb”).

In a second step, the data processing consisted of identifying the foot-on and heel-off events from the data of the IMUs positioned on the cannon bone.

The cannon bone *x*-axis acceleration signal showed repeatable peaks between strides and between horses, during all the stance phases, corresponding to foot-on and heel-off moments (Figure 3). All strides were windowed between each maximum peak of the cannon bone *y*-axis gyroscope, using the method described in [8], corresponding to the swing phase of each stride, allowing to focus on the studied stance phase in the middle of the processing window.

In the same way as the reference events, according to the exercise, the ground condition and limb timing of the *i*th step of the foot-on and heel-off were labeled ImuCannonFootOn (*i*, “exercise”, “ground”, “limb”) and ImuCannonHeelOff (*i*, “exercise”, “ground”, “limb”).

#### 2.3.1. Foot-On

**Figure 4:**
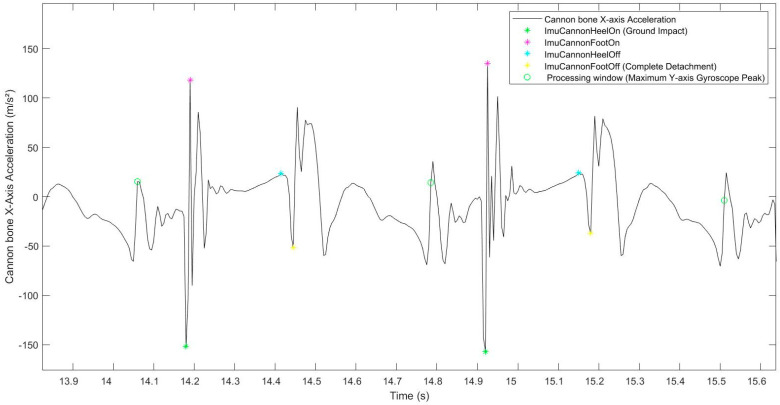
Representation of the cannon bone *x*-axis acceleration signal used to detect the foot-on (magenta points) and heel-off (cyan points) events.

In the processing window, the first positive peak was identified, corresponding to the ImuCannonFootOn (*i*, “Line”, “ground”, “limb”), detected with a threshold of 85% of the maximum value of the cannon bone *x*-axis acceleration. This peak of ImuCannonFootOn (*i*, “exercise”, “ground”, “limb”) always follows a negative peak, corresponding to the hoof ground impact (Figure 4).

In a circle (CircleIn and CircleOut exercise), on all types of ground (ImuCannonFootOn (*i*, “Circle”, “ground”, “Fore”)), the foot-on event was identified by the last positive peak before the plateau, detected with a threshold of 85% of the maximum value of the cannon bone *x*-axis acceleration in the processing window.

#### 2.3.2. Heel-Off

In the processing window, the first positive peak, after the plateau was identified, corresponding to the ImuCannonHeelOff (*i*, “exercise”, “ground”, “limb”) (Figure 4). The peak of ImuCannonHeelOff (*i*, “exercise”, “ground”, “limb”) was followed by a negative peak, corresponding to the foot-off event (complete hoof lift-off), itself detected with a threshold of 85% of the maximum value of the cannon bone *x*-axis acceleration.

**Figure 5:**
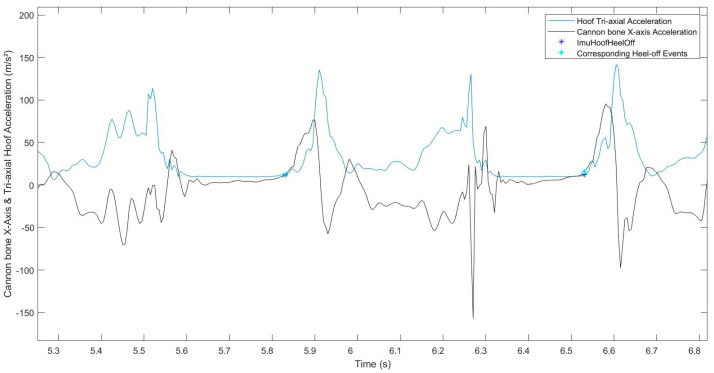
Representation of the hoof tri-axial acceleration (blue curve) and the cannon bone *x*-axis acceleration (black curve), for the “forelimb inside the circle” condition. The blue points correspond to the reference heel-off, and the cyan points correspond to the detected heel-off (from the cannon bone IMU data). Unlike all the other conditions, the cannon bone *x*-axis acceleration shows only one peak at the foot-off moment.

For the ImuCannonHeelOff (*i*, “CircleIn”, “ground”, “Fore”), the curve did not always follow the same curve shape as in other conditions. The signal sometimes showed only one large positive peak after the plateau (Figure 5). The ImuCannonHeelOff (*i*, “CircleIn”, “ground”, “Fore”) was obtained by detecting a change of curve variation from a derivative subsampled signal, with the Piecewise Cubic Hermite Interpolating Polynomial Matlab function. All calculations were performed by self-made functions with Matlab software (Matlab R2021a, The MathWorks Inc., Natick, MA, USA).

For each condition and both limbs, the stride duration and stance duration were calculated with these equations:

Stride Duration ImuCannon (*i*) = ImuCannonFootOn (*i* + 1) − ImuCannonFootOn (*i*), (1)

Stance Duration ImuCannon (*i*) = ImuCannonHeelOff (*i*) − ImuCannonFootOn (*i*), (2)

Stride Duration ImuHoof (*i*) = ImuHoofFootOn (*i* + 1) − ImuHoofFootOn (*i*), (3)

Stance Duration ImuHoof (*i*) = ImuHoofHeelOff (*i*) − ImuHoofFootOn (*i*), (4)

The stance duration was normalized with the stride duration to obtain information on the percentage of stride duration, with the following equation:

Stance Duration (*i*)% stride duration = (Stance Duration (*i*)/Stride Duration (*i*)) × 100, (5)

#### 2.3.3. Additional Method

An additional method, taken from the literature [9], was also investigated. This method uses the cannon bone tri-axial acceleration to detect foot-on (hoof completely laid flat on the ground) (AdMethodFootOn (*i*, “straight line”, “ground”, “limb”)) and foot-off (complete hoof lift-off) (AdMethodFootOff (*i*, “straight line”, “ground”, “limb”)) events on the straight line. First, the raw data were segmented into rough periods from midstance to midstance. To identify rough locations of the swing and stance periods, a median filter with a window length of half the sampling frequency followed by a second-order Butterworth filter with a 5 Hz and 20 Hz cut-off frequency was applied to the forelimb and hindlimb cannon data, respectively. The cannon bone tri-axial acceleration was then filtered with a second-order Butterworth filter with a 40 Hz cut off frequency. This method identifies the foot-on and foot-off events, respectively, by the last prominent peak before the stance phase and by the first prominent peak after the stance phase, in the processing window.

### 2.4. Statistical Analysis

All the foot-on and heel-off events (ImuCannonFootOn (*i*, “exercise”, “ground”, “limb”) and ImuCannonHeel-Off (*i*, “exercise”, “ground”, “limb”)), detected from the cannon bone IMU, were compared with the reference events, the foot-on and heel-off, obtained from the hoof IMU (ImuHoofFootOn (*i*, “exercise”, “ground”, “limb”) and ImuCannonHeel-Off (*i*, “exercise”, “ground”, “limb”)).

The precision and accuracy of foot-on and heel-off detection, as well as the stance duration, were studied with Bland–Altman’s method [23,24,25] for evaluating the agreement among the two measurements techniques (developed method with IMU on the cannon bone and reference method with IMU on the hoof). With this method, each of the n samples is represented on a graph by assigning the mean of the two measurements as the x-value, and the difference between the two measurements as the y-value.

The Bland–Altman’s method is described by these equations:

Difference = developed method − reference method(6)

Bias = mean (difference)(7)

SD = standard deviation (difference)(8)

LimitAgreementHigh = bias + 1.96 × SD(9)

LimitAgreementLow = bias − 1.96 × SD(10)

The accuracy, i.e., bias (Equation (7)), was defined by the mean difference between the developed methods values and reference method values. The precision was defined as the standard deviation of the differences (SD) (Equation (8)). The limits of agreement (Equations (9) and (10)) were also calculated, corresponding to the range including 95% of the differences values were. All the results were expressed as a percentage of the stride duration.

The foot-on (AdMethodFootOn (*i*, “straight line”, “ground”, “limb”)) and foot-off (AdMethodFootOff (*i*, “straight line”, “ground”, “limb”)) events, obtained with the additional method, were compared with the reference events, the foot-on and heel-off, obtained from the hoof IMU (ImuHoofFootOn (*i*, “exercise”, “ground”, “limb”) and ImuCannonHeel-Off (*i*, “exercise”, “ground”, “limb”)), for the straight line on hard ground only. The results expressed in the study [9] did not allow us to compare other exercises or other grounds. The precision and accuracy of this method were also studied with Bland–Altman’s method [23,24,25]. These results were compared with previously published results and with results from our developed methods. In this part, the results were expressed in milliseconds (ms).

## 3. Results

Stride durations, calculated from each foot-on of the equipped forelimbs, are shown in Table 1.

For the foot-on detection, the precision and accuracy of the developed method are shown in Table 2. Whatever the condition, the average bias was less than 1.79% of the stride duration for the forelimb and 3.45% of the stride duration for the hindlimb.

For the heel-off detection, the precision and accuracy of the developed method are shown in Table 3. Whatever the condition, the average bias was less than 1.46% of the stride duration for the forelimb and 1.94% of the stride duration for the hindlimb.

For the stance phase duration, the precision and accuracy of the developed method are shown in Table 4. Whatever the condition, the average bias was less than −1.16% of stride duration for the forelimb and −3.22% of the stride duration for the hindlimb.

The results of comparison between our method and the tested additional method from [9], in a straight line on hard ground, are shown in Table 5. The foot-on detection results showed similar bias, with no difference for the forelimb and a 7 ms difference for the hindlimb. The heel-off detection was different between the two tested methods, with a 46 ms difference for the forelimb and 43 ms difference for the hindlimb.

For all these results (Table 2, Table 3, Table 4 and Table 5), the Bland–Altman graphs are available in the supplementary data attached document.

## 4. Discussion

In this study, an innovative data processing method was developed to detect foot-on and heel-off events of horse locomotion, based on a single IMU located on the cannon bone of the horse, for several conditions and several surfaces.

Previous study has shown a bias of less than 0.6% of the stride duration for foot-on detection and 0.1% of the stride duration for heel-off detection, for forelimbs, at the trot on treadmill, based on cannon bone IMU data sampling at 500 Hz [8]. At trot, on hard ground in a straight line, our method of foot-on and heel-off detection demonstrates a bias lower than 0.4% of stride duration for foot-on and 1.4% of stride duration for heel-off, based on IMU data sampling at 200 Hz. At this sampling rate, there are 5 milliseconds (ms) between two frames, which is the measurement precision of our system. Consequently, the bias got from our developed method are equivalent to bias of, respectively, 0.6 frames (3 ms) for foot-on and 2.2 frames (11 ms) for heel-off, with a mean stride duration of 159 frames (795 ms).

Our method has the advantage of being applicable to various conditions and surfaces, and it shows a mean bias of −0.4% (percentage of stride duration) for the forelimb stance duration and a mean bias of −1.5% (percentage of stride duration) for the hindlimb stance duration. In a previous study [8], the strides were recorded only on a treadmill, involving a stabilized gait and more repeatable strides between themselves. In our study, horses trotted in real conditions, involving an unsteady gait with variable speeds and strides.

We noticed that the right circle (equipped limb inside the circle) on hard ground, for the hindlimb, induced a larger bias for the foot-on detection than the other conditions, with a bias of 3.45% of stride, which appears to be relatively constant given the standard deviation of 1.09% of stride. The explanation could be found because the reference foot-on was deduced by the hoof tri-axial gyroscope data, which measures not only the rotational movement of the limb but also the additional rotation movements due to the circle displacement of the horse. In addition, the cannon bone and the hoof are separated by different joints and segment, especially the metacarpophalangeal joint (MPJ). The MPJ mainly allows flexion/extension movements in the sagittal plane and small-amplitude movements in axial rotation and abduction/adduction [26]. The hindlimb had a role of propulsion in the gait, and a joint movement dissociation is observable, with closing of the hip and rapid opening of the other joint angles to ensure the foot-on event [27]. Consequently, during circle exercises, the combination of the whole horse rotation in addition to the horse’s hindlimb rotation could alter the gyroscope data curve shape and shift the detection of the foot-on, inducing a possible bias in our reference foot-on. Moreover, methods using gyroscopic signals are known to drift over time [28], which can affect the measurement capability.

Compared to the method developed by Briggs and Mazza on the straight-line condition only [9], our method shows similar results. For foot-on detection on the forelimb, our results showed 3 ± 13 ms bias, and the results obtained by Briggs and Mazza showed 3 ± 6 ms bias [9]. For foot-on detection on the hindlimb, the bias was −4 ± 9 ms with our method and −3 ± 22 ms with the Briggs and Mazza method. These converging results seem to demonstrate the ability of cannon-bone-mounted IMUs to accurately detect the foot-on events. Concerning the heel-off detection, the discrepancies between the Briggs and Mazza method and our method were larger, with 46 ms of absolute deviation for the forelimb and 43 ms of absolute deviation for the hindlimb. These differences could be explained by the difference in the reference events for both methods. Indeed, in our method, we focused on the detection of the heel-off event, corresponding to the take-off of the heel, while Briggs and Mazza seem to focus on the foot-off event, corresponding to the complete hoof lift-off. Therefore, these differences could be expected, and it should be stressed that both events, heel-off and foot-off, have clinical interest and are complementary.

However, this study presents some limitations. First, only seven sound horses of trotting and thoroughbred breeds were included in the study, with an average of 42 ± 4 strides recorded (mean ± SD) per horse. This small amount of collected data did not present a large population diversity, and further investigations could be focused to test these methods on a larger horse population as well as on lame horses, or on other horse breeds. Other limitations also concern the reference events. According to [10,11], the reference events were detected manually on hoof gyroscope or acceleration, which can involve inter-examiner bias. In addition, this method [10,11] was tested in laboratory conditions with hard ground only, while it was assumed that this method held for all ground surfaces and all conditions.

To conclude, this study presents an original method, developed to detect stride events for different conditions (straight line and circle) with different surfaces (hard and soft ground) and based on the *x*-axis acceleration of the horse’s cannon bone. Even if our study is only based on the trot gait, we are confident that our approach could be used as a guideline to adapt or test methods on other gaits. As perspectives, the use of a single versatile IMU on the horse’s cannon bone could allow simplifying the horse locomotion analysis. The study of the stance duration, with the use of a single IMU and in field conditions, in healthy and lame horses could allow to further develop the knowledge in equine locomotion. The plans for future works would be to adapt and validate these gait event detection methods on other gaits, especially walking and galloping. In the future, it would allow us to characterize the complete horse locomotion during a locomotor examination or training, as well as to monitor its global health condition.

## Figures and Tables

**Table 1:** Mean stride durations (mean) and standard deviations (SD) were indicated for each condition: straight line (SL), left circle (LC), and right circle (RC), for both ground conditions—hard (H) and soft (S)—and for the right forelimb and right hindlimb. For the left-circle condition, the equipped limb was the one outside the circle. For the right-circle condition, the equipped limb was the one inside the circle. Results are in millisecond.

Condition	Mean	SD
SL H	685 ms	31 ms
SL S	720 ms	33 ms
LC H	742 ms	32 ms
RC H	733 ms	40 ms
LC S	754 ms	38 ms
RC S	749 ms	37 ms

**Table 2:** Bland–Altman comparison (in percentage of stride duration%) of the reference foot-on event and the foot-on event detected with the developed method from the cannon bone data. Accuracy (bias), precision (SD), and upper/lower limits of agreement (ULA and LLA) were indicated for each condition: straight line (SL), left circle (LC), and right circle (RC), for both ground conditions—hard (H) and soft (S)—and for the right forelimb and right hindlimb. For the left-circle condition, the equipped limb was the one outside the circle. For the right-circle condition, the equipped limb was the one inside the circle. Results are in percentage of stride duration.

Condition	Right Forelimb				Right Hindlimb			
	Bias	SD	ULA	LLA	Bias	SD	ULA	LLA
SL H	0.40%	1.87%	4.06%	−3.26%	0.59%	1.40%	3.33%	−2.15%
SL S	1.79%	1.65%	5.01%	−1.44%	0.88%	1.36%	3.54%	−1.78%
LC H	0.96%	1.72%	4.34%	−2.42%	0.75%	0.72%	2.18%	−0.67%
RC H	1.12%	2.02%	5.07%	−2.83%	3.45%	1.09%	5.59%	1.32%
LC S	0.15%	3.74%	7.48%	−7.17%	−0.56%	0.50%	0.42%	−1.53%
RC S	1.14%	2.43%	5.90%	−3.63%	−0.12%	0.73%	1.31%	−1.54%

**Table 3:** Bland–Altman comparison (in percentage of stride duration%) of the reference foot-off event and the foot-off event detected with the developed method from the cannon bone data. Accuracy (bias), precision (SD), and upper/lower limits of agreement (ULA and LLA) were indicated for each condition: straight line (SL), left circle (LC), and right circle (RC), for both ground conditions—hard (H) and soft (S)—and for the right forelimb and right hindlimb. For the left-circle condition, the equipped limb was the one outside the circle. For the right-circle condition, the equipped limb was the one inside the circle. Results are in percentage of stride duration.

Condition	Right Forelimb				Right Hindlimb			
	Bias	SD	ULA	LLA	Bias	SD	ULA	LLA
SL H	1.40%	1.68%	4.70%	−1.89%	−0.25%	1.65%	2.98%	−3.48%
SL S	1.46%	1.80%	4.99%	−2.08%	−0.20%	1.34%	2.42%	−2.83%
LC H	−0.19%	1.68%	3.10%	−3.48%	−0.95%	1.69%	2.36%	−4.25%
RC H	0.08%	2.81%	5.58%	−5.42%	0.24%	3.54%	7.18%	−6.70%
LC S	0.16%	1.77%	3.62%	−3.30%	−1.94%	1.17%	0.35%	−4.22%
RC S	0.38%	2.33%	4.96%	−4.20%	−0.74%	2.13%	3.44%	−4.92%

**Table 4:** Bland–Altman comparison (in percentage of stride duration%) of the reference stance duration and stance duration obtained with the developed method from the cannon bone data. Accuracy (bias), precision (SD), and upper/lower limits of agreement (ULA and LLA) were indicated for each condition: straight line (SL), left circle (LC), and right circle (RC), for both ground conditions—hard (H) and soft (S)—and for the right forelimb and right hindlimb. For the left-circle condition, the equipped limb was the one outside the circle. For the right-circle condition, the equipped limb was the one inside the circle. Results are in percentage of stride.

Condition	Right Forelimb				Right Hindlimb			
	Bias	SD	ULA	LLA	Bias	SD	ULA	LLA
SL H	1.02%	2.44%	5.80%	−3.77%	−0.84%	1.69%	2.47%	−4.15%
SL S	−0.34%	1.82%	3.22%	−3.90%	−1.09%	1.05%	0.96%	−3.14%
LC H	−1.16%	2.36%	3.46%	−5.78%	−1.71%	1.85%	1.92%	−5.33%
RC H	−1.04%	3.37%	5.56%	−7.64%	−3.22%	3.63%	3.89%	−10.33%
LC S	−0.28%	2.11%	3.87%	−4.42%	−1.38%	1.25%	1.07%	−3.82%
RC S	−0.76%	3.17%	5.45%	−6.97%	−0.62%	2.38%	4.05%	−5.28%

**Table 5:** Bland–Altman comparison (in milliseconds) of the reference foot-on and heel-off events and the foot-on and heel-off events detected with the method from Briggs and Mazza [9] and our method from the cannon bone data. Accuracy (bias), precision (SD), and upper/lower limits of agreement (ULA and LLA) were indicated for the straight-line-on-hard-ground condition, and for the right forelimb and right hindlimb. For both events (foot-on and heel-off), the table shows results obtained from the same dataset with our method (Hatrisse & al.) and the Briggs and Mazza method [9].

Event	Method	Right Forelimb				Right Hindlimb			
		Bias	SD	ULA	LLA	Bias	SD	ULA	LLA
Foot-on	Hatrisse & al.	3 ms	13 ms	27 ms	−22 ms	4 ms	9 ms	22 ms	−14 ms
	Briggs and Mazza	3 ms	6 ms	16 ms	−9 ms	−3 ms	22 ms	40 ms	−46 ms
Heel-off	Hatrisse & al.	10 ms	13 ms	35 ms	−15 ms	−2 ms	12 ms	22 ms	−25 ms
	Briggs and Mazza	−36 ms	51 ms	64 ms	−137 ms	−45 ms	31 ms	16 ms	−106 ms

## Data Availability

Further data is contained in the supplementary material section of this paper. Any additional requests can be addressed to the corresponding author.

## Supplementary Materials

The following supplementary data can be downloaded at: https://www.mdpi.com/article/10.3390/s22030703/s1.
