# Supplementary material for: Stance Phase Detection by Inertial Measurement Unit Placed on the Metacarpus of Horses Trotting on Hard and Soft Straight Lines and Circles
Source: Sensors (Basel). 2022 Jan 18;22(3):703. doi: 10.3390/s22030703 (PMC8840150; doi:10.3390/s22030703)
Supplement: Supplementary file 1 [file sensors-22-00703-s001.zip › sensors-1516709-supplementary.pdf]

Supplementary data

Following **Table 2**, here are the Bland-Altman graphs of the reference Foot-on event and the Foot-on event detected with the developed method from the canon bone data. Accuracy (Bias), precision (SD) and Upper/Lower Limits of Agreement (ULA & LLA) were represented for Straight Line and Hard ground condition (SL H) for forelimbs on graph A and hindlimbs on graph B, Straight Line and Soft ground condition (SL S) for forelimbs on graph C and hindlimbs on graph D, Left Circle and Hard ground condition (LC H) for forelimbs on graph E and hindlimbs on graph F, Right Circle and Hard ground condition (RC H) for forelimbs on graph G and hindlimbs on graph H, Left Circle and Soft ground condition (LC S) for forelimbs on graph I and hindlimbs on graph J and Right Circle and Hard ground condition (RC S) for forelimbs on graph K and hindlimbs on graph L.

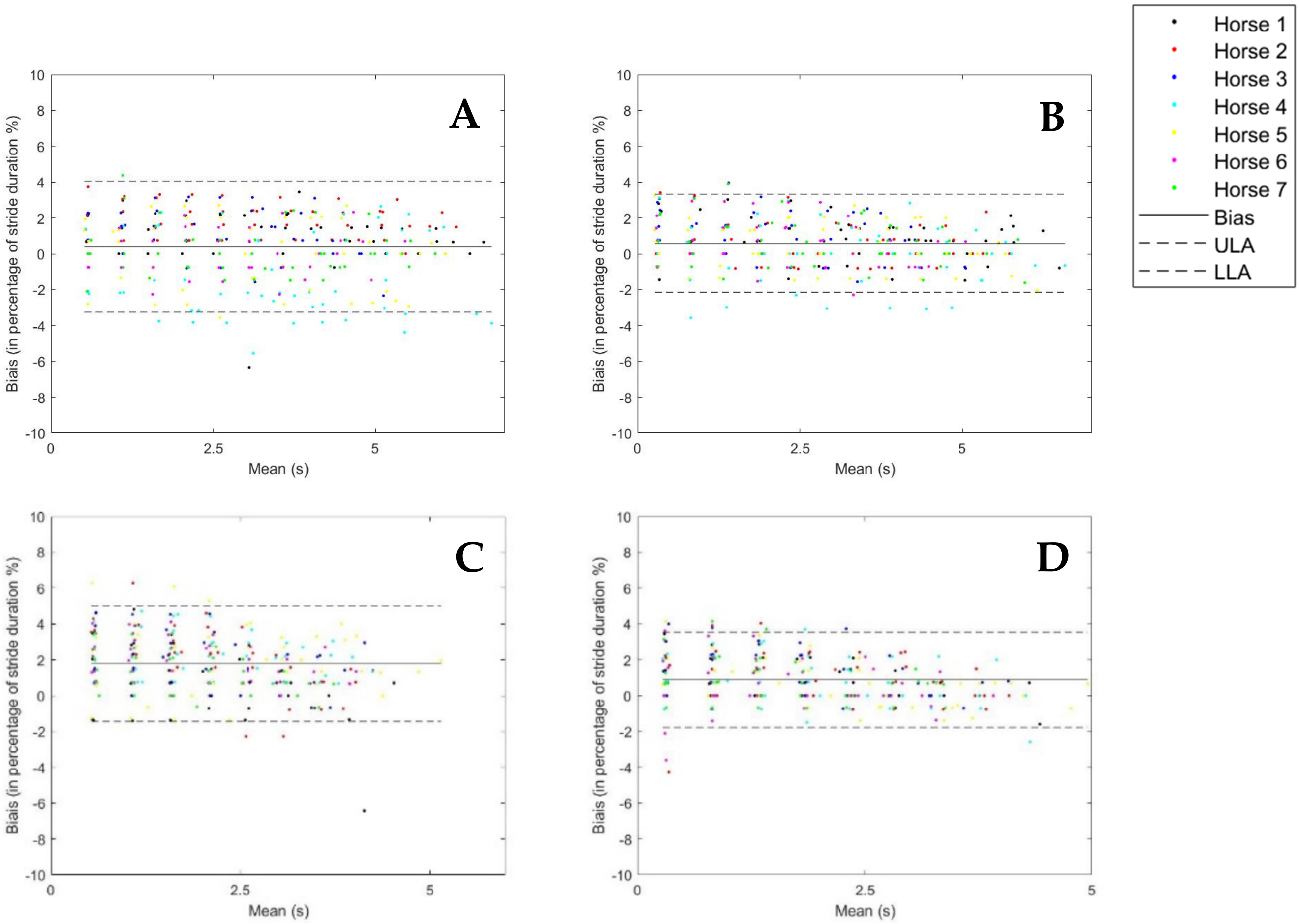

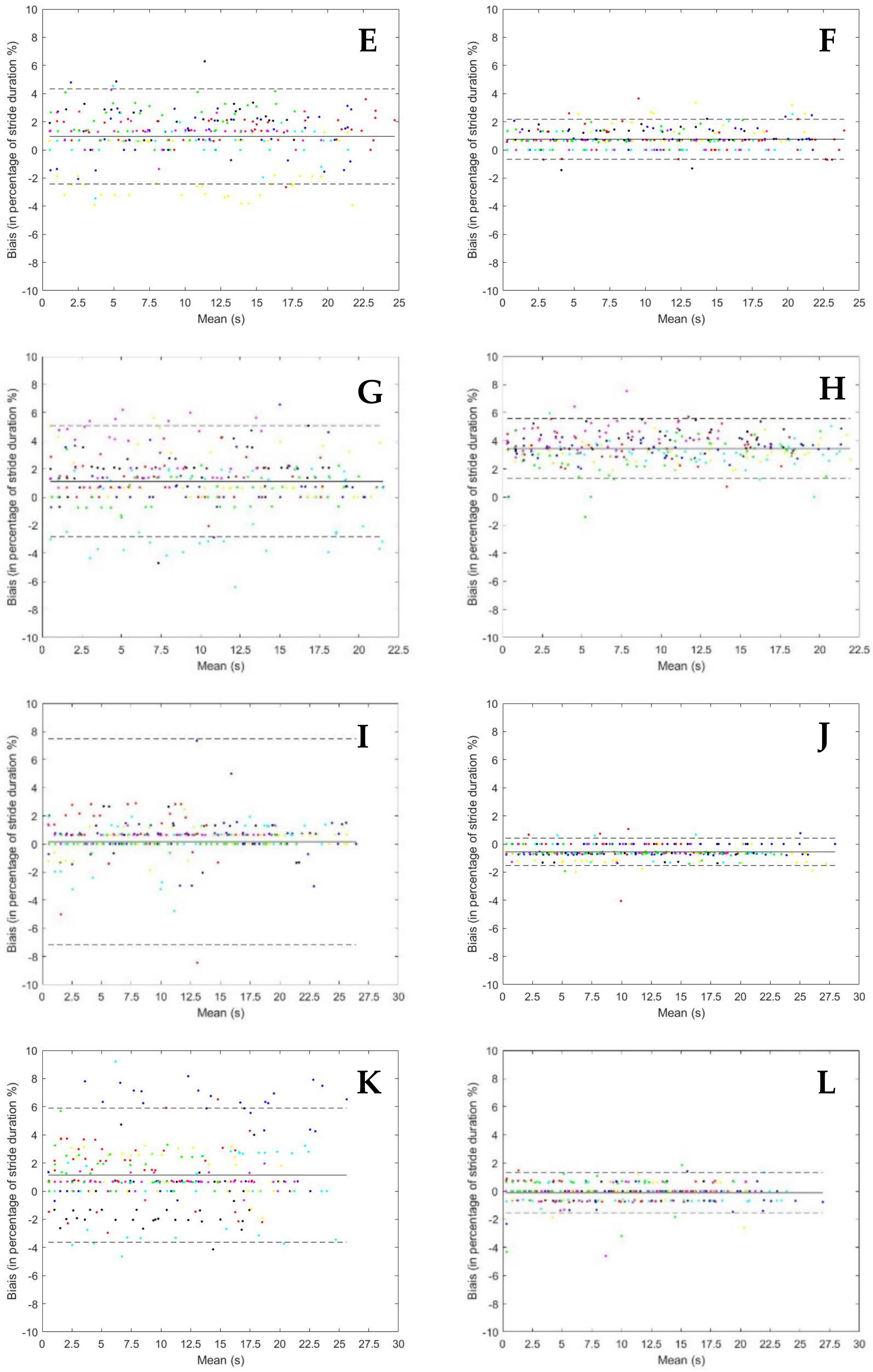
E
F
G
H
I
J
K
L
Biais (in percentage of stride duration %)
Mean (s)

Following **Table 3**, here are the Bland-Altman graphs of the reference Foot-off event and the Foot-off event detected with the developed method from the canon bone data. Accuracy (Bias), precision (SD) and Upper/Lower Limits of Agreement (ULA & LLA) were represented for Straight Line and Hard ground condition (SL H) for forelimbs on graph A and hindlimbs on graph B, Straight Line and Soft ground condition (SL S) for forelimbs on graph C and hindlimbs on graph D, Left Circle and Hard ground condition (LC H) for forelimbs on graph E and hindlimbs on graph F, Right Circle and Hard ground condition (RC H) for forelimbs on graph G and hindlimbs on graph H, Left Circle and Soft ground condition (LC S) for forelimbs on graph I and hindlimbs on graph J and Right Circle and Hard ground condition (RC S) for forelimbs on graph K and hindlimbs on graph L.

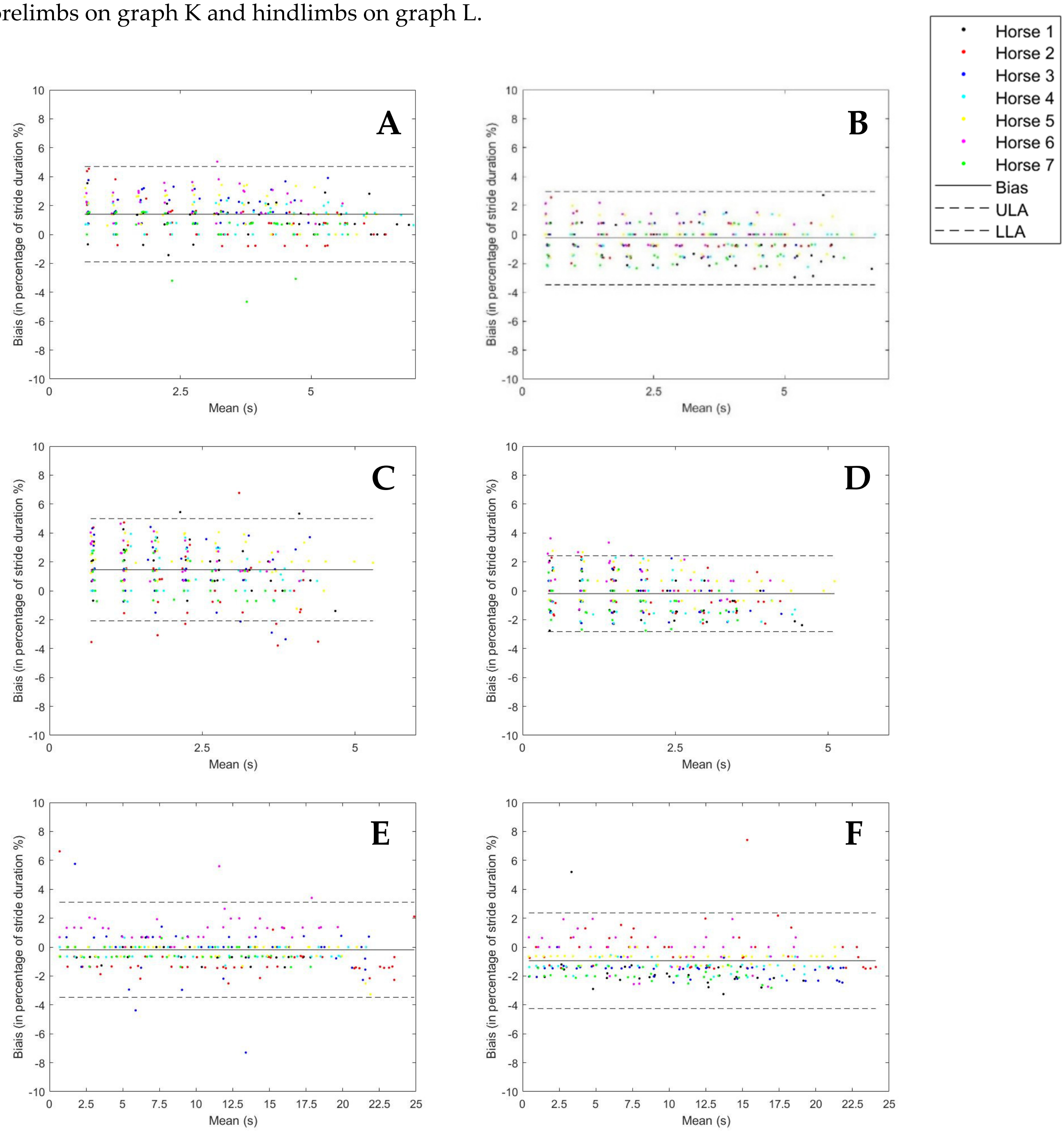

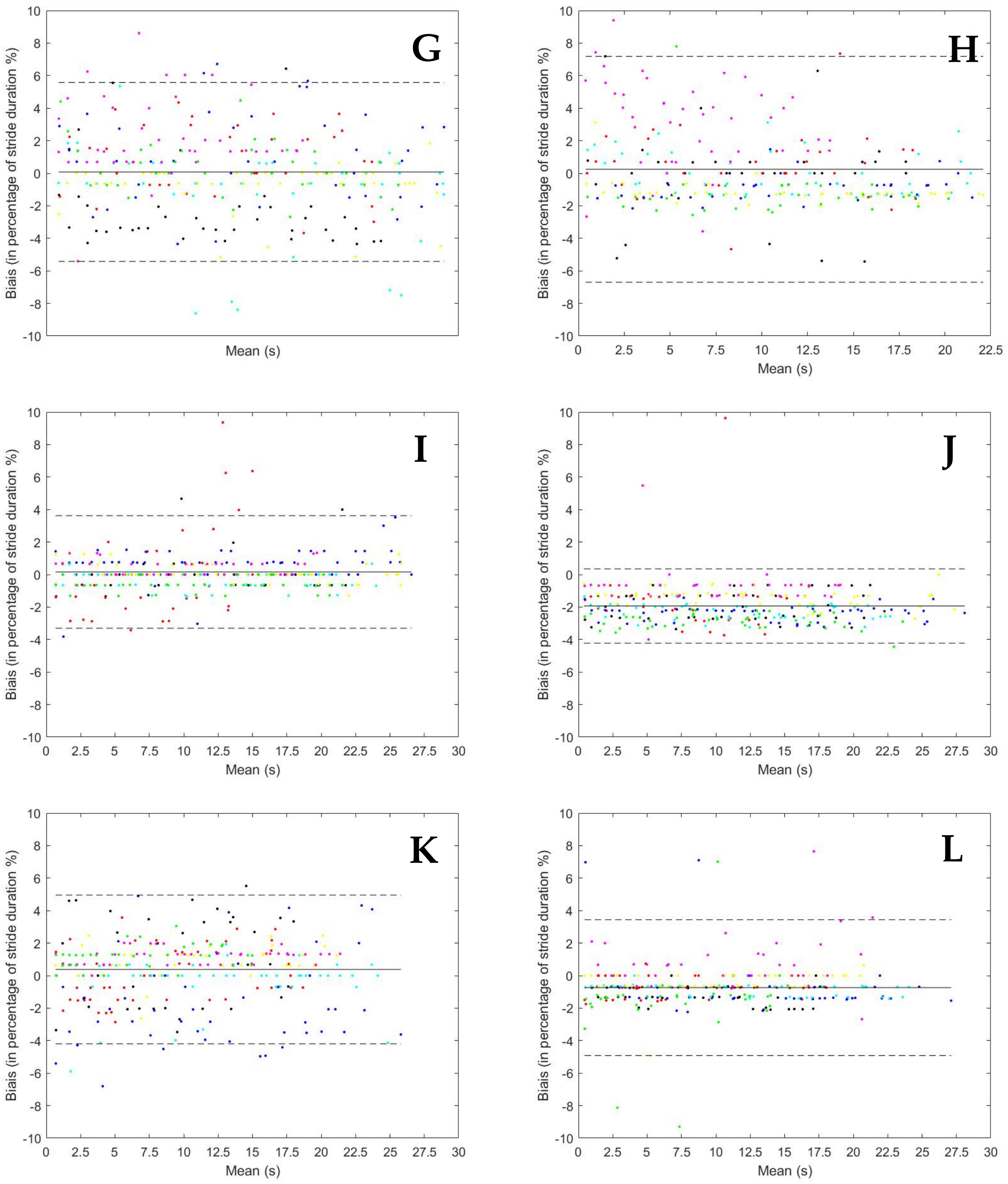
G
H
I
J
K
L
Biais (in percentage of stride duration %)
Mean (s)

Following **Table 4**, here are the Bland-Altman graphs of the reference Stance Duration and the Stance Duration got with the developed method from the canon bone data. Accuracy (Bias), precision (SD) and Upper/Lower Limits of Agreement (ULA & LLA) were represented for Straight Line and Hard ground condition (SL H) for forelimbs on graph A and hindlimbs on graph B, Straight Line and Soft ground condition (SL S) for forelimbs on graph C and hindlimbs on graph D, Left Circle and Hard ground condition (LC H) for forelimbs on graph E and hindlimbs on graph F, Right Circle and Hard ground condition (RC H) for forelimbs on graph G and hindlimbs on graph H, Left Circle and Soft ground condition (LC S) for forelimbs on graph I and hindlimbs on graph J and Right Circle and Hard ground condition (RC S) for forelimbs on graph K and hindlimbs on graph L.

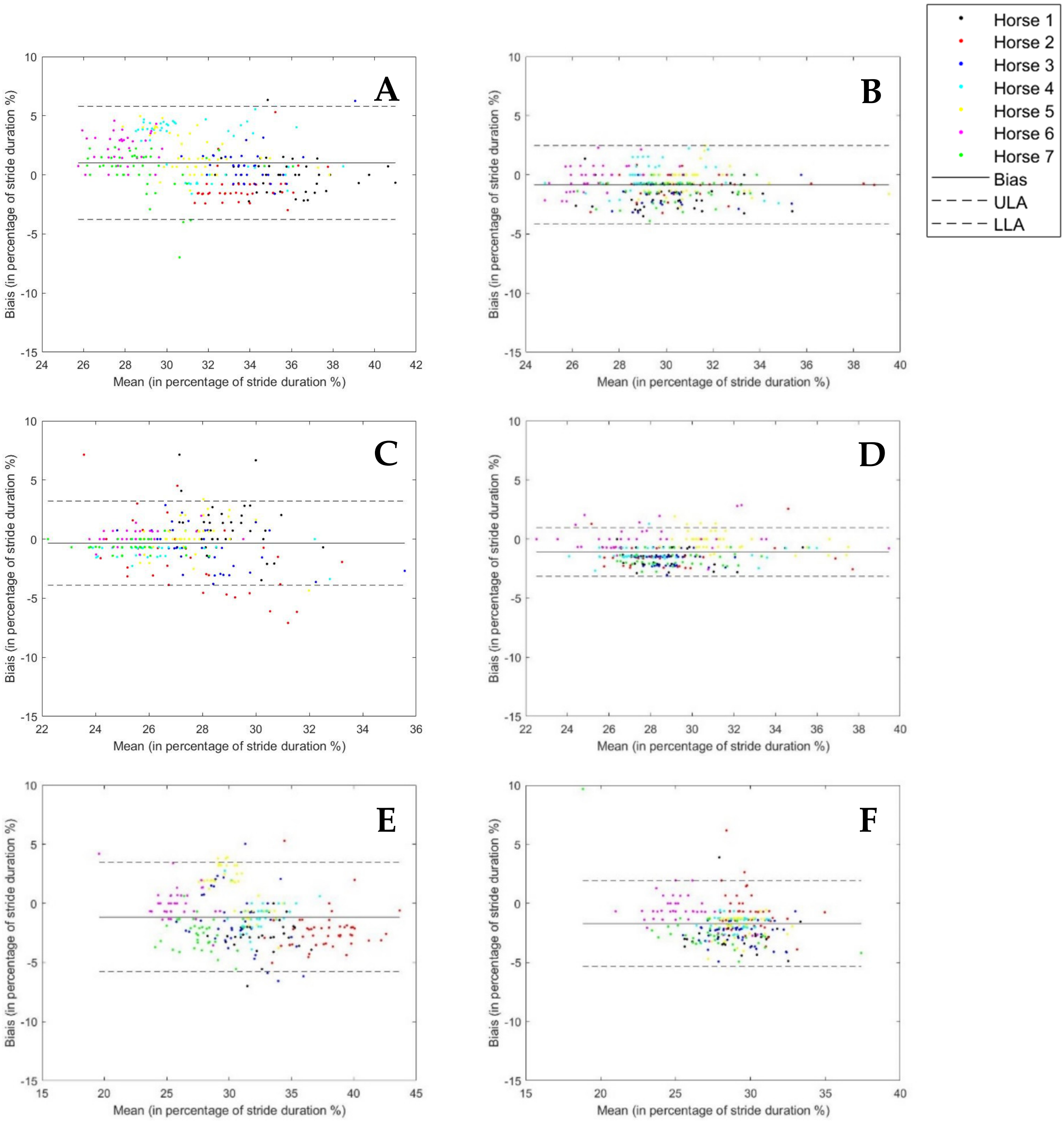

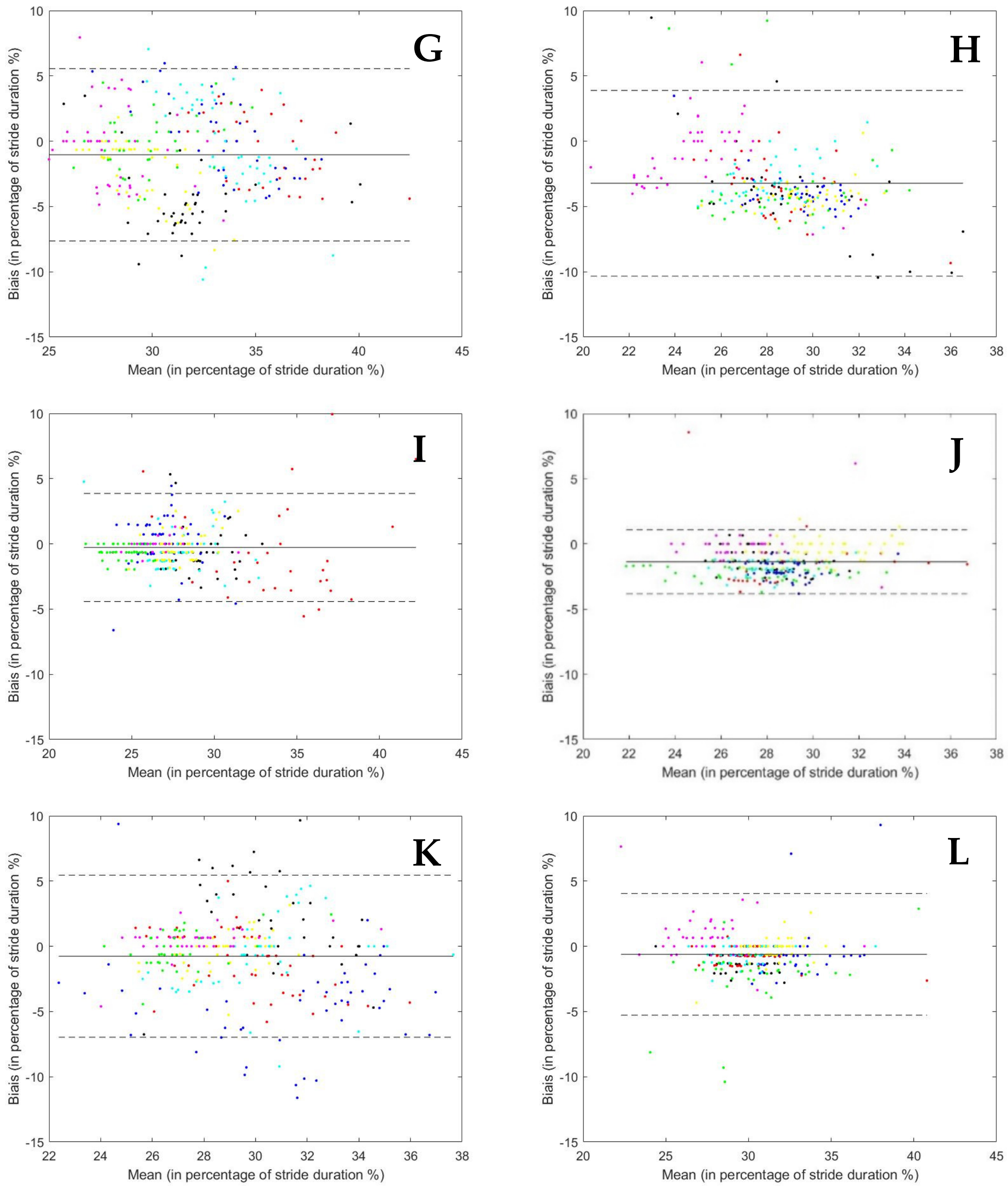
G
H
I
J
K
L
Biais (in percentage of stride duration %)
Mean (in percentage of stride duration %)

Following **Table 5**, here are the Bland-Altman graphs of the reference Foot-on and Heel-off events and the Foot-on and Heel-off events detected with the method from Briggs & Mazza [11] and our method. Accuracy (Bias), precision (SD) and Upper/Lower Limits of Agreement (ULA & LLA) were represented for the straight line on hard ground condition. The graph A represent the Foot-on statistics, detected on the right forelimb from the Briggs & Mazza method. The graph B represent the Foot-on statistics, detected on the right hindlimb from the Briggs & Mazza method. The graph C represent the Foot-on statistics, detected on the right forelimb from our method. The graph D represent the Foot-on statistics, detected on the right hindlimb from our method. And in the same way for Heel-off, the graph E represent the Heel-off statistics, detected on the right forelimb from the Briggs & Mazza method. The graph F represent the Heel-off statistics, detected on the right hindlimb from the Briggs & Mazza method. The graph G represent the Heel-off statistics, detected on the right forelimb from our method. The graph H represent the Heel-off statistics, detected on the right hindlimb from our method.

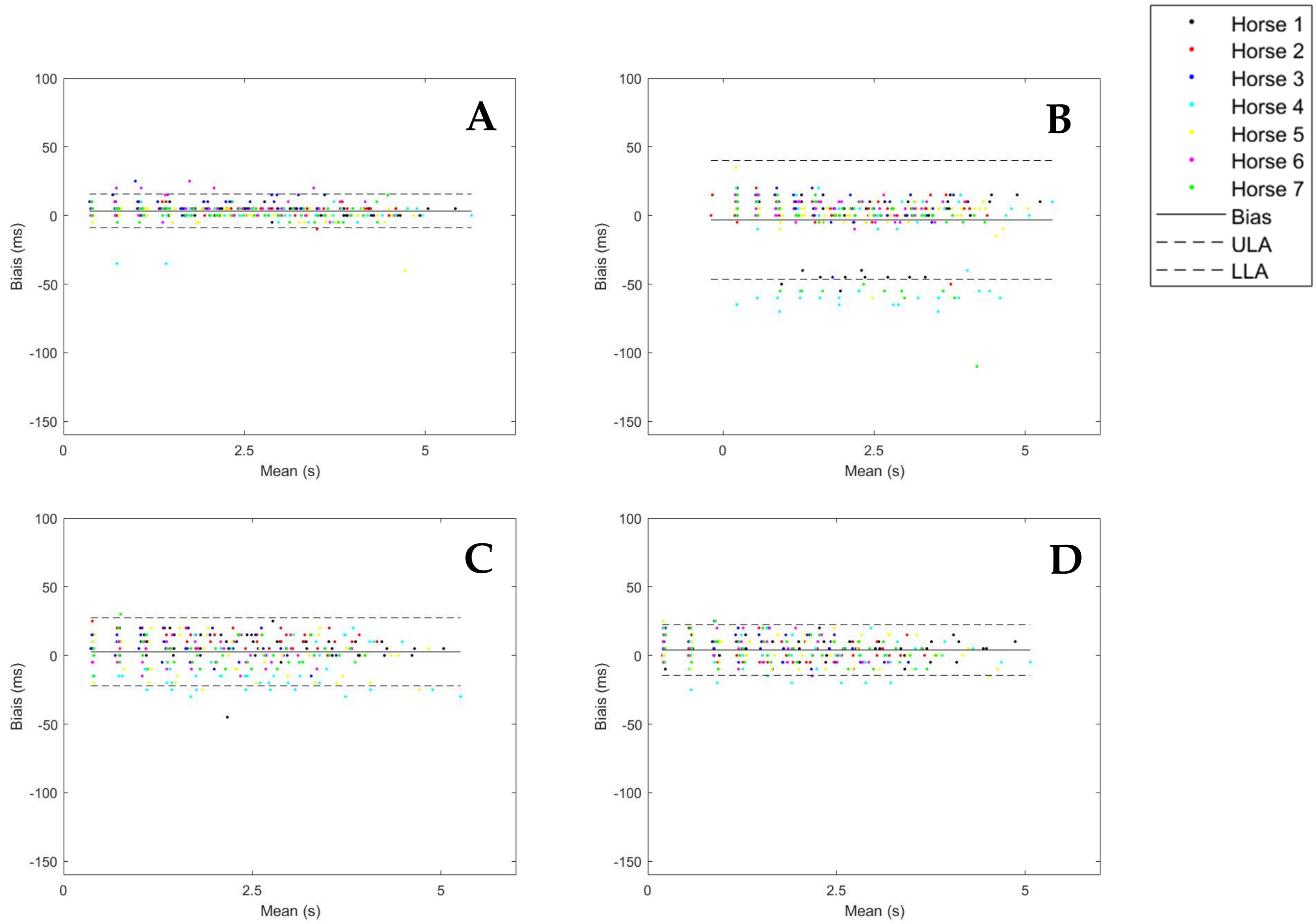

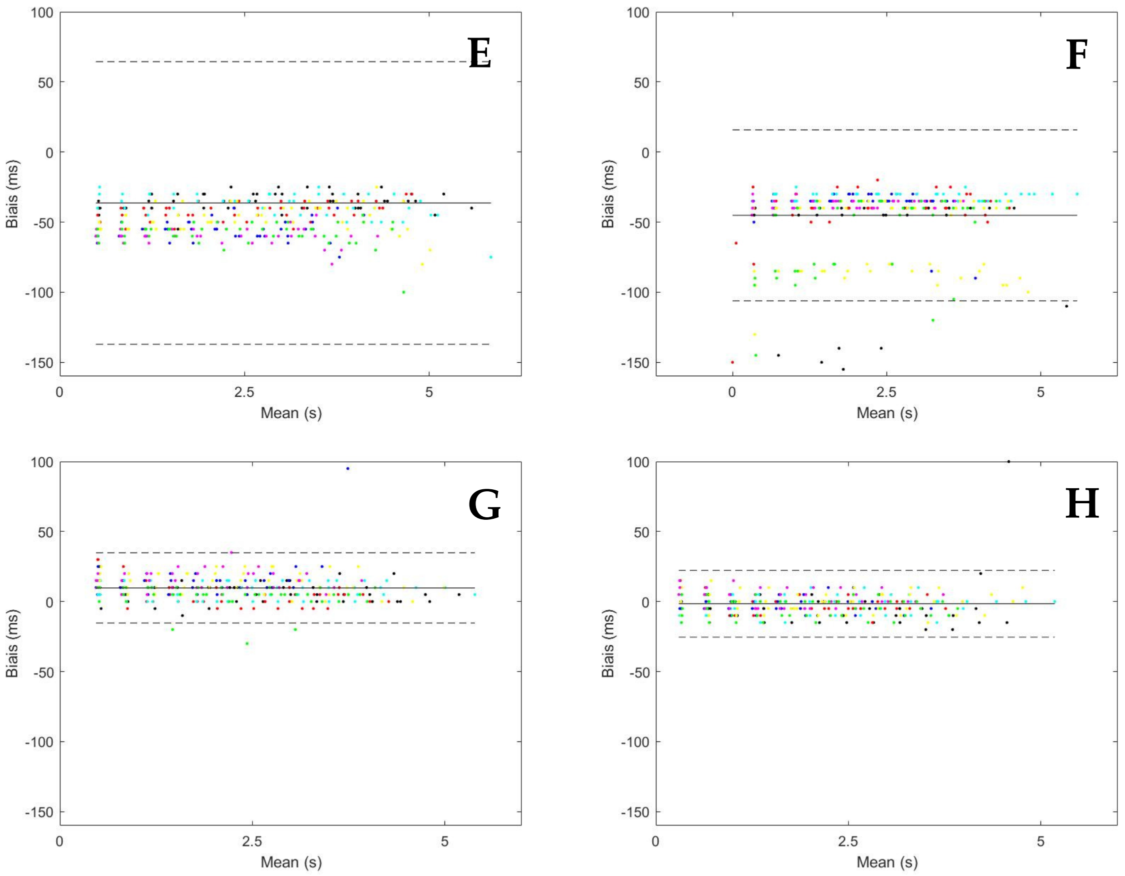
E
F
G
H
Biais (ms)
Mean (s)
100
50
0
-50
-100
-150
0
2.5
5
